# A novel clinical prediction model of severity based on red cell distribution width, neutrophil-lymphocyte ratio and intra-abdominal pressure in acute pancreatitis in pregnancy

**DOI:** 10.1186/s12884-023-05500-0

**Published:** 2023-03-18

**Authors:** Wenyan Liao, Guangwei Tao, Guodong Chen, Jun He, Chunfen Yang, Xiaohua Lei, Shuo Qi, Jiafeng Hou, Yi Xie, Can Feng, Xinmiao Jiang, Xin Deng, Chengming Ding

**Affiliations:** 1 The First Affiliated Hospital, Department of Gynaecology and Obstetrics, Hengyang Medical School, University of South China, Hengyang, Hunan 421001 China; 2 The First Affiliated Hospital, Department of Hepatopancreatobiliary Surgery, Hengyang Medical School, University of South China, No. 69, Chuanshan Road, Hengyang, Hunan 421001 China; 3 The Nanhua Affiliated Hospital, Hengyang Medical School, University of South China, Hengyang, Hunan 421001 China

**Keywords:** Acute pancreatitis in pregnancy, Inflammation, Red cell distribution width, Neutrophil-lymphocyte ratio, Intra-abdominal pressure, Prediction model

## Abstract

**Background:**

Acute pancreatitis in pregnancy (APIP) with a high risk of death is extremely harmful to mother and fetus. There are few models specifically designed to assess the severity of APIP. Our study aimed to establish a clinical model for early prediction of severity of APIP.

**Methods:**

A retrospective study in a total of 188 patients with APIP was enrolled. The hematological indicators, IAP (intra-abdominal pressure) and clinical data were obtained for statistical analysis and prediction model construction.

**Results:**

According to univariate and multivariate logistic regression analysis, we found that red cell distribution width (RDW), neutrophil-lymphocyte ratio (NLR) and Intra-abdominal pressure (IAP) are prediction indexes of the severity in APIP (*p*-value < 0.05). Our novel clinical prediction model was created by based on the above three risk factors and showed superior predictive power in primary cohort (AUC = 0.895) and validation cohort (AUC = 0.863). A nomogram for severe acute pancreatitis in pregnancy (SAPIP) was created based on the three indicators. The nomogram was well-calibrated.

**Conclusion:**

RDW, NLR and IAP were the independent risk factors of APIP. Our clinical prediction model of severity in APIP based on RDW, NLR and IAP with predictive evaluation is accurate and effective.

## Introduction

Acute pancreatitis in pregnancy (APIP) is a severe disease that affects 1 in 1,000 to 12,000 pregnant women on average, which is more frequently than the general population [1]. Both the mother and the fetus might suffer substantial morbidity as a result of APIP. The APIP still has high rates of maternal and perinatal mortality, 3.3% and 11.6–18.7%, respectively [1].

Acute pancreatitis (AP) is an inflammatory disorder that can damage nearby and distant organs even result in the multiple organ dysfunction. According to the current Atlanta classification based on the presence and duration of organ failure (2012), acute pancreatitis is divided into three types mild acute pancreatitis (MAP), moderately severe acute pancreatitis (MSAP), and severe acute pancreatitis (SAP) [2]. Patients without organ dysfunction and local complications were MAP. Patients with temporary organ dysfunction (≤ 48 h) and/or localized or systemic accompanying diseases were classified as MSAP. SAP is persistent organ dysfunction (> 48 h). The risk of maternal and fetal death is highly associated with the severity of APIP [1, 3]. Reports indicate that SAP in pregnancy (SAPIP) with the maternal mortality rate as high as approximately 20–40% is serious hazardous to the health of pregnant women [4, 5]. Early recognition of APIP severity is critically important for prompt treatment to individual patients. The early stage of AP is usually referred to the first week after the disease onset [6]. Early prediction of the severity of APIP is very important for clinical treatment.

Elevated internal pressure (IAP) is common in critically ill patients. Intraperitoneal hypertension (IAH) has adverse effects on hemodynamics, respiration, and renal function, and may eventually lead to multiple organ failure [7]. It is reported that early recognition of IAH may help in early intervention improving outcomes of acute necrotizing pancreatitis [8]. However, to our knowledge, few studies have been conducted on IAP in APIP.

Currently, several prediction systems are usually used for AP patients. However, the prediction system is not specific for pregnant women, and some do not apply in pregnant women. At present, there is few scoring systems designed for patients with APIP in clinical practice [9]. Therefore, it is very urgent and important to establish a timely, simple and useful clinical prediction model to predict severe acute pancreatitis in pregnancy.

Red cell distribution width (RDW) is a parameter primarily reflecting the volume variability of red blood cells. Recent many studies indicated that RDW relates with the levels of many kinds of inflammatory cytokines in serum [10]. Moreover, RDW has been introduced as a novel inflammatory predictor in various diseases, such as functional bowel conditions [11], autoimmune diseases [12], rheumatoid arthritis [13], degenerative vertebral conditions [14], autoimmune hepatitis [15]. It is proved that RDW can be used to predict mortality and severity of patients with AP [16, 17]. However, few studies focused on the prediction of RDW in the severity of patients with APIP. On the other hand, neutrophil-lymphocyte ratio (NLR) has been introduced as a marker of inflammation in many inflammatory related diseases, such as inflammatory bowel disease [18], diabetes mellitus [19], thyroiditis [20], and AP where NLR can be used to predicting severity [21]. Up to date, it is not clear whether NLR and RDW can be used to construct predictive model of APIP severity. Therefore, we included RDW and NLR in our study, aiming to further determine their predictive role in APIP severity and whether a predictive model of APIP severity can be established.

In this study, 188 cases of APIP patients were retrospectively reviewed and classified into two groups by us. MAP and MSAP were included into non-SAPIP groups, and severe acute pancreatitis in pregnancy was included into SAPIP groups. we collected routine laboratory tests data within 48 h after the APIP onset, IAP and other clinical data to assess the predictive ability of these data on the severity of APIP, in order to construct a clinical prediction model of severity in APIP.

## Methods

### Patients selection

**Fig. 1:**
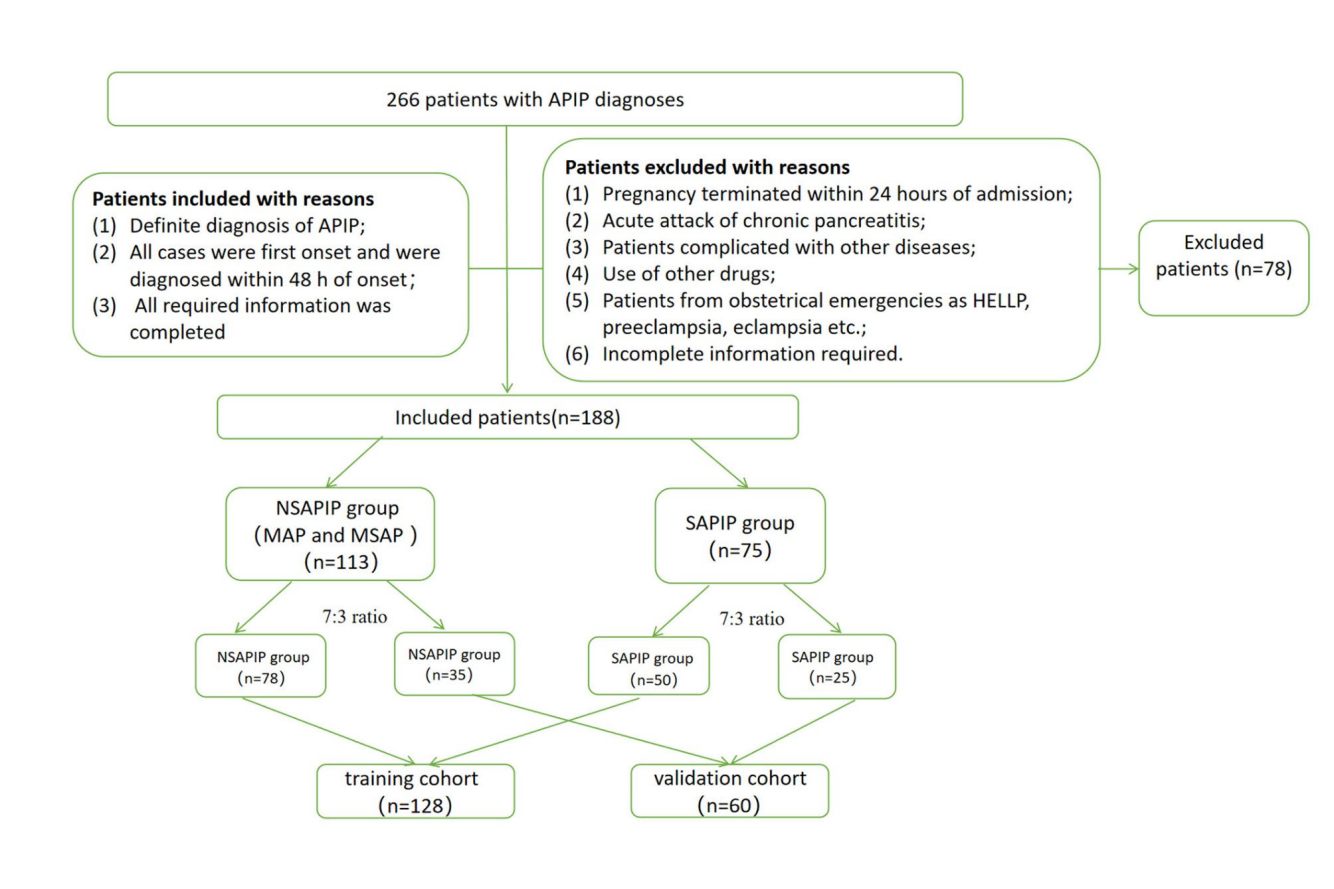
The selection process for patients in a flow chart

The medical records of patients who were diagnosed with APIP were retrospectively collected at our hospital (The First Affiliated Hospital of University of South China) from January 2008 to December 2021. Patients meeting the following criteria were included: (1) Definite diagnosis of APIP; (2) All cases were first onset and were diagnosed within 48 h of onset. (3) All required information was completed. Exclusion criteria: (1) Pregnancy terminated within 24 h of admission; (2) Acute attack of chronic pancreatitis; (3) Patients complicated with other diseases, such as malignant tumor, comorbidities related to pregnancy or not related to pregnancy, sepsis, hemorrhagic disease (trauma, for example), other Inflammation-related disease, and so on; (4) Use of immunosuppressants, corticosteriod and other drugs; (5) Patients received red blood cells; (7) Patients from obstetrical emergencies as HELLP, preeclampsia, eclampsia etc.; (8) Incomplete information required. The flowchart prensented in Fig. 1. The Ethics Committee of our hospital approved our study, and our study was carried out following the Declaration of Helsinki.

### Data collection

We collected the following clinical parameters: age, gestational weeks, red cell distribution width (RDW), alanine aminotransferase (ALT), Albumin, triglyceride (TG), total cholesterol (TC), blood urea nitrogen (BUN), serum creatinine (Scr), Calcium, lactate dehydrogenase (LDH), intra-abdominal pressure (IAP), neutrophil–lymphocyte ratio (NLR). All clinical parameters were tested in the hospital and were collected within 48 h of admission. Among them, hematological test results were obtained on the day of admission, and IAP values were measured within 48 h after admission, the average values of the two highest IAP values were taken, and pressure was measured indirectly through the bladder [22].

### Definitions

We diagnosed the APIP patients via the 2012 revised version of the Atlanta criteria [6], the patients were diagnosed with APIP, if they met more than 2 pieces out of the following criteria: (1) characteristic abdominal pain of AP; (2) serum amylase or lipase was more than 3 times of the normal upper limit value; (3) characteristic results of acute pancreatitis from cross-sectional abdominal imaging. We graded the severity of APIP basing on 2012 revised version of the Atlanta criteria, and patients without organ dysfunction and local complications were mild acute pancreatitis (MAP). Patients with temporary organ disorder (≤ 48 h) and/or local or systemic other diseases caused by AP were moderately severe acute pancreatitis (MSAP). Patients with persistent organ dysfunction (> 48 h) were severe acute pancreatitis (SAP). MAP and MSAP were included into NSAPIP group, and severe pancreatitis in pregnancy was included into SAPIP group.

### Development and validation of the prediction model

**Fig. 2:**
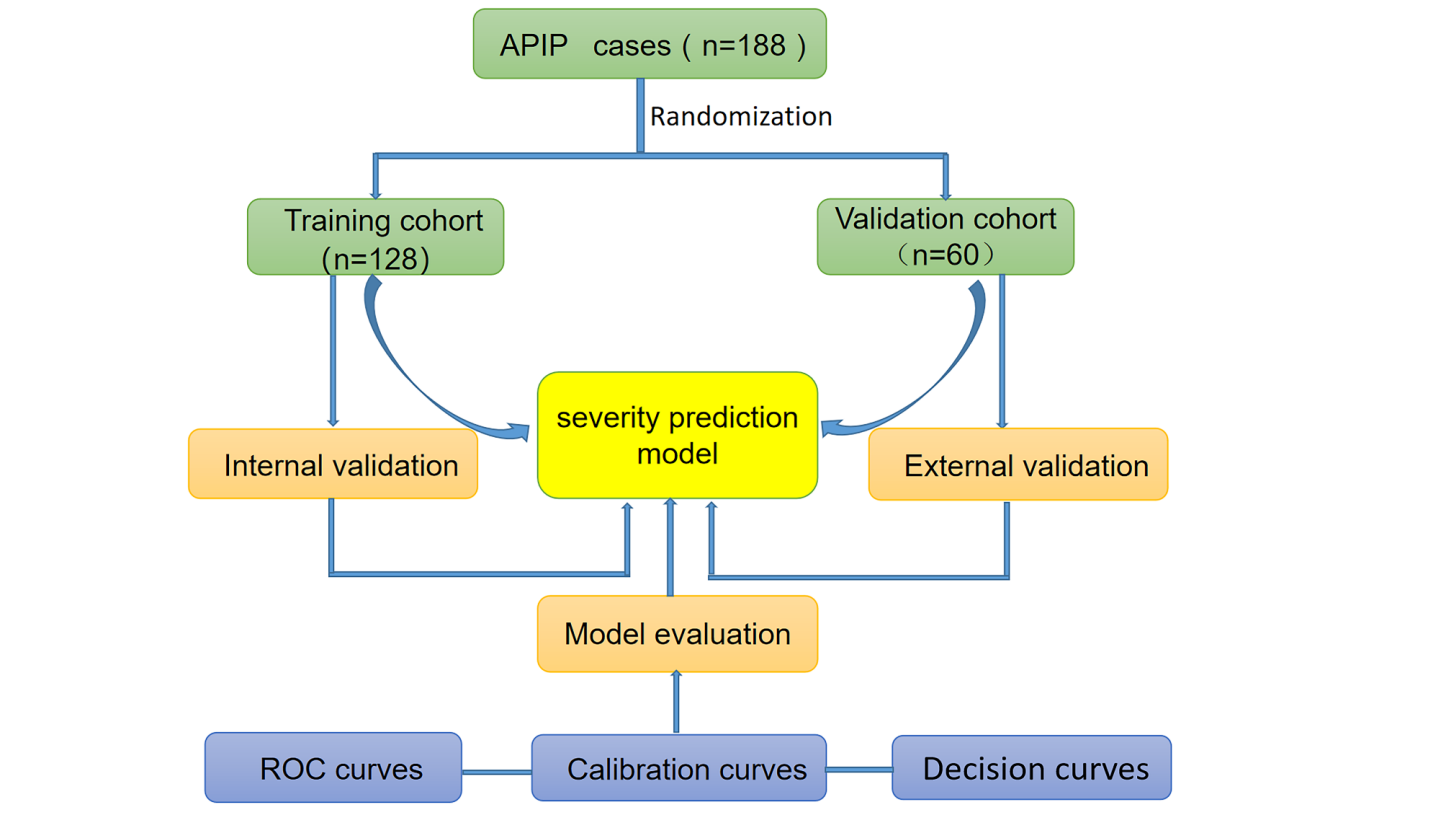
Research flow chart

Variables were screened by univariate logistic regression analysis. To establish our prediction model, we collected all associated factors to carry out the multivariate logistic regression analysis. We evaluated the accuracy of independent prediction factors in the predictive model of SAPIP by the receiver operating characteristic (ROC) curves. A nomogram for SAPIP was produced according to the multivariate logistic regression model. To validate the consistency and accuracy of the model in predicting severity of APIP patients, we applied the internal and external validation sets to assess the consistency, and used the calibration curves, ROC curves to check the accuracy of the prediction model in this study. In the end, decision analysis was applied to further assess the clinical applicability of the predictive model. Decision curve analysis (DCA) was carried out to evaluate the clinical utility of the prediction model. The flowing chart presents in Fig. 2.

### Statistical analysis

We presented all the variables as mean ± standard deviation (SD) or median (range), as appropriate. Statistical analyses were conducted by GraphPad Prism 7.0 Software for Windows (GraphPad Software, La Jolla, CA, USA), Service Solutions SPSS Software 25.0 (SPSS, Chicago, IL, USA) and R statistical software (version 4.2.0; https://www.r-project.org/ ). Kolmogorov–Smirnov test was used for normality analysis of the study variables. Student’s t-test was applied to analyse normally distributed continuous variables, and the Mann-Whitney U test was utilized to analyse nonnormally distributed continuous variables. Univariate logistic regression analysis was performed to identify predictive factors of APIP. Predictive factors with *p* value less than 0.05 in univariate analysis were included in the multivariate analysis. Multivariate logistic regression analysis was conducted to identify independent predictive factors, and check the useful combination of factors that could predict APIP. All *p* values were two-sided, with statistical significance set at *p* values less than 0.05.

## Results

### Basic characteristics of the patients between training cohort and validation cohort

A total of 188 APIP patients were enrolled, and a 7:3 ratio of training cohort (n = 128) to validation cohort (n = 60) were randomly allocated. The detailed baseline characteristics of APIP patients in this investigation were presented in Table 1.

**Table 1:** Baseline Characteristics of APIP Patients Between Training Cohort and Validation Cohort

Variable	Training cohort (n = 128)	Validation cohort (n = 60)	t/Z	*p*
Age(years)	29(22–42)	29(22–41)	-0.761	0.447
Gestational weeks	30.19 ± 4.26	30.22 ± 3.07	-0.053	0.958
RDW(%)	14.1(10.9–19.1)	13.55(11.1–18.2)	-1.001	0.317
ALT(U/L)	17.15(6.8-318.5)	17.85(6.8-300.4)	-0.122	0.903
Albumin(g/L)	31.1(23.6–42)	31.9(26.1–42.1)	-1.856	0.063
BUN(mmol/L)	5.35(2.2–18.6)	6.2(2.5–17.9)	-1.822	0.068
TG(mmol/L)	3.58(1.28-131.57)	3.69(1.6–57.6)	-0.016	0.987
TC(mmol/L)	6.22(2.2–32.8)	5.66(2.68-31)	-0.522	0.602
Scr(umol/L)	69(26–282)	72(27–262)	-0.295	0.768
Calcium(mmol/L)	1.95(1.2–2.9)	1.99(1.25–2.9)	-1.314	0.189
LDH(U/L)	342(103–1435)	340(177–1214)	-0.066	0.947
IAP(mmHg)	8.3(5.4–13.7)	9.35(5.5–13.8)	-0.938	0.348
NLR	13.60 ± 3.64	14.18 ± 3.35	-1.045	0.298

### Clinical characteristics between NSAPIP and SAPIP groups

**Fig. 3:**
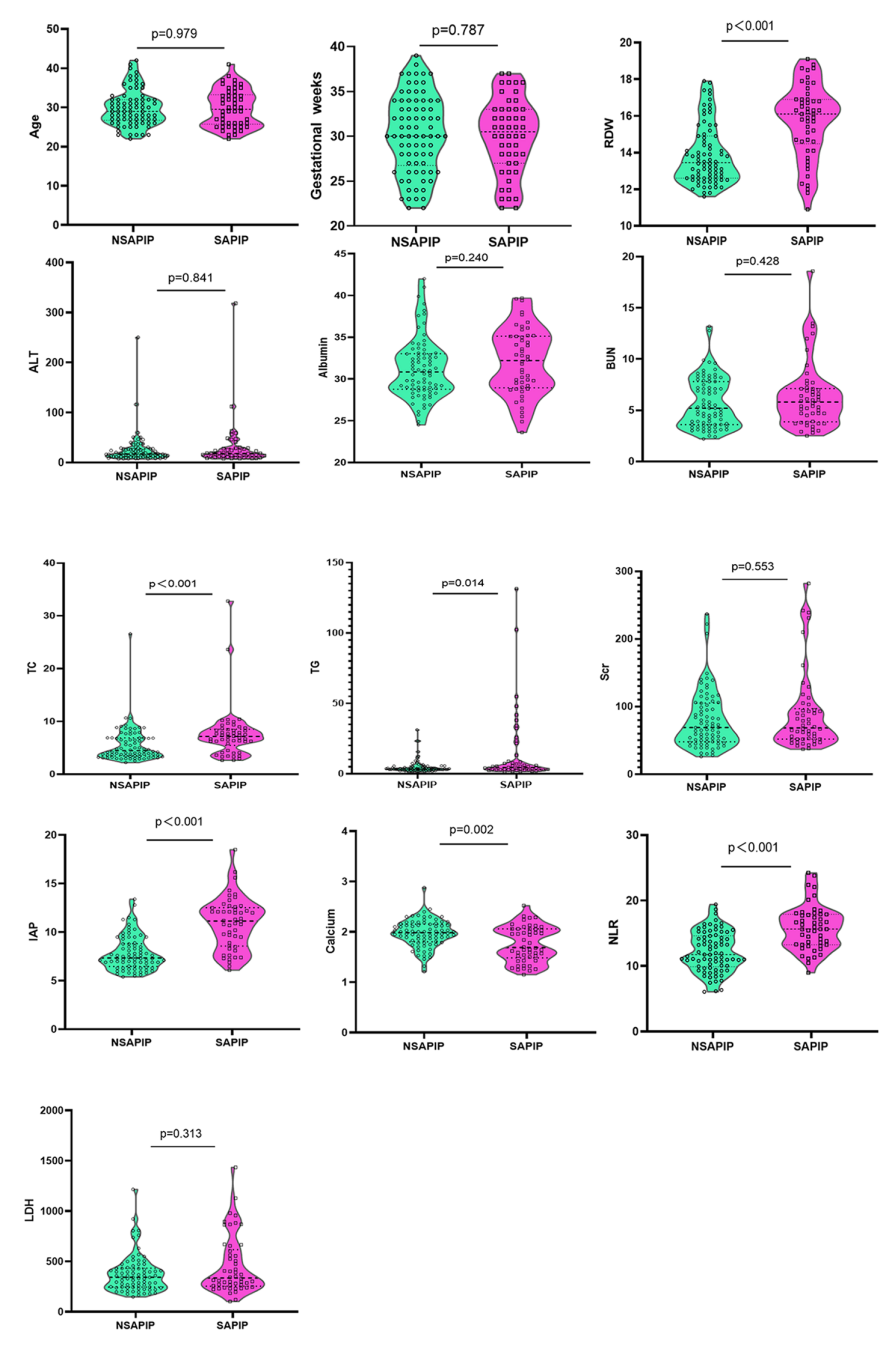
Violin plots of clinical characteristics between NSAPIP and SAPIP Groups

As shown in Fig. 3, we compared and analysed the general conditions between the two groups. Significant differences in some variables were found between NSAPIP and SAPIP groups, including: RDW, TC, TG, Calcium, NLR, IAP(*p*<0.05). No significant differences in age, gestational weeks, ALT, albumin, BUN, Scr, and LDH were found between the two groups of patients.

### Univariate and multivariate logistic regression analysis

**Fig. 4:**
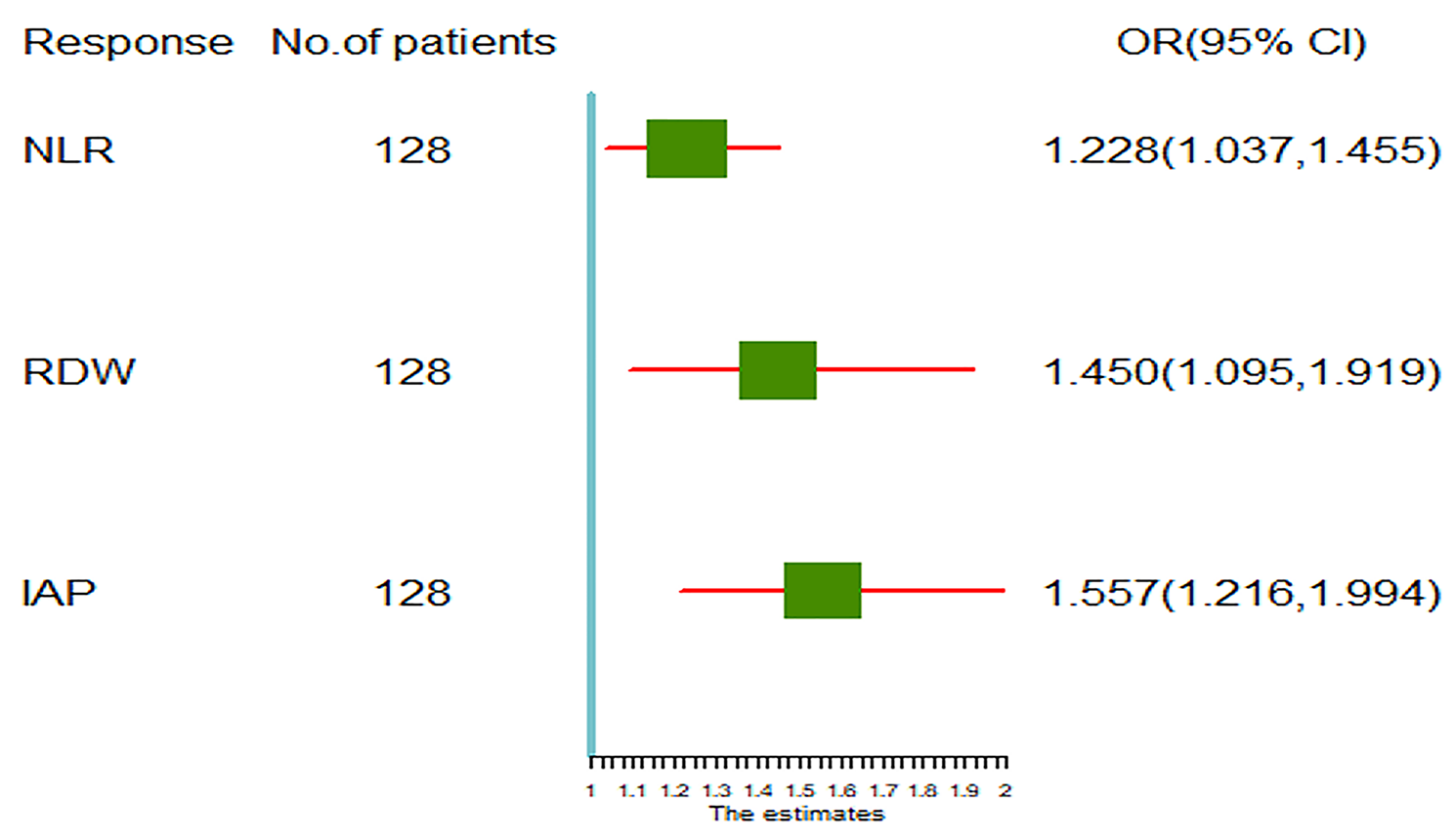
Forest plot of independent predictors of SAPIP with odds-ratio

As shown in Table 2, the univariate logistic regression analysis indicated that the patients’ RDW [odds-ratio (OR) = 1.683, p = 0.000], TG (OR = 1.072, p = 0.007), TC(OR = 1.182, p = 0.017), IAP(OR = 1.827, p = 0.000), calcium(OR = 0.108, p = 0.001) and NLR(OR = 1.434, p = 0.000) were candidate factors related to the predicting severity of APIP. What’s more, the results of univariate logistic regression analysis indicated that age, ALT, albumin, BUN, Scr, and LDH were not severity predictors of APIP. This result was consistent with the result of variables comparison between NSAPIP and SAPIP groups. These factors which were not severity predictors of APIP were thus excluded from the multivariate logistic regression analysis.The results of the multivariate logistic regression analysis were showed as follows : RDW(OR = 1.450, p = 0.009), IAP(OR = 1.557,p = 0.000), NLR (OR = 1.228, p = 0.017). These results meant that RDW, IAP, NLR are independent prediction marker of severity in APIP patients. Moreover, we also constructed a forest plot of independent predictors of SAPIP with odds-ratio (Fig. 4).

**Table 2:** Univariable and multivariate logistic regression analyses of factors for severity prediction of APIP in the training cohor (*p < 0.05)

Variable	Univariate analysis			Multivariate analysis		
	OR	95%CI	*p*	OR	95%CI	*p*
Age	0.999	0.924–1.079	0.975			
Gestational weeks	0.978	0.918–1.043	0.504			
RDW	1.683	1.353–2.094	0.000*	1.450	1.095–1.919	0.009*
ALT	1.003	0.994–1.013	0.499			
Albumin	1.058	0.963–1.163	0.239			
BUN	1.088	0.956–1.238	0.204			
TG	1.072	1.019–1.128	0.007*	1.056	0.974–1.144	0.185
TC	1.182	1.030–1.355	0.017*	1.122	0.971–1.297	0.119
Scr	1.004	0.997–1.011	0.278			
Calcium	0.108	0.031–0.381	0.001*	0.288	0.053–1.569	0.150
LDH	1.001	1.000-1.003	0.059			
IAP	1.827	1.477–2.260	0.000*	1.557	1.216–1.994	0.000*
NLR	1.434	1.241–1.659	0.000*	1.228	1.037–1.455	0.017*

### Model construction and validation

**Fig. 5:**
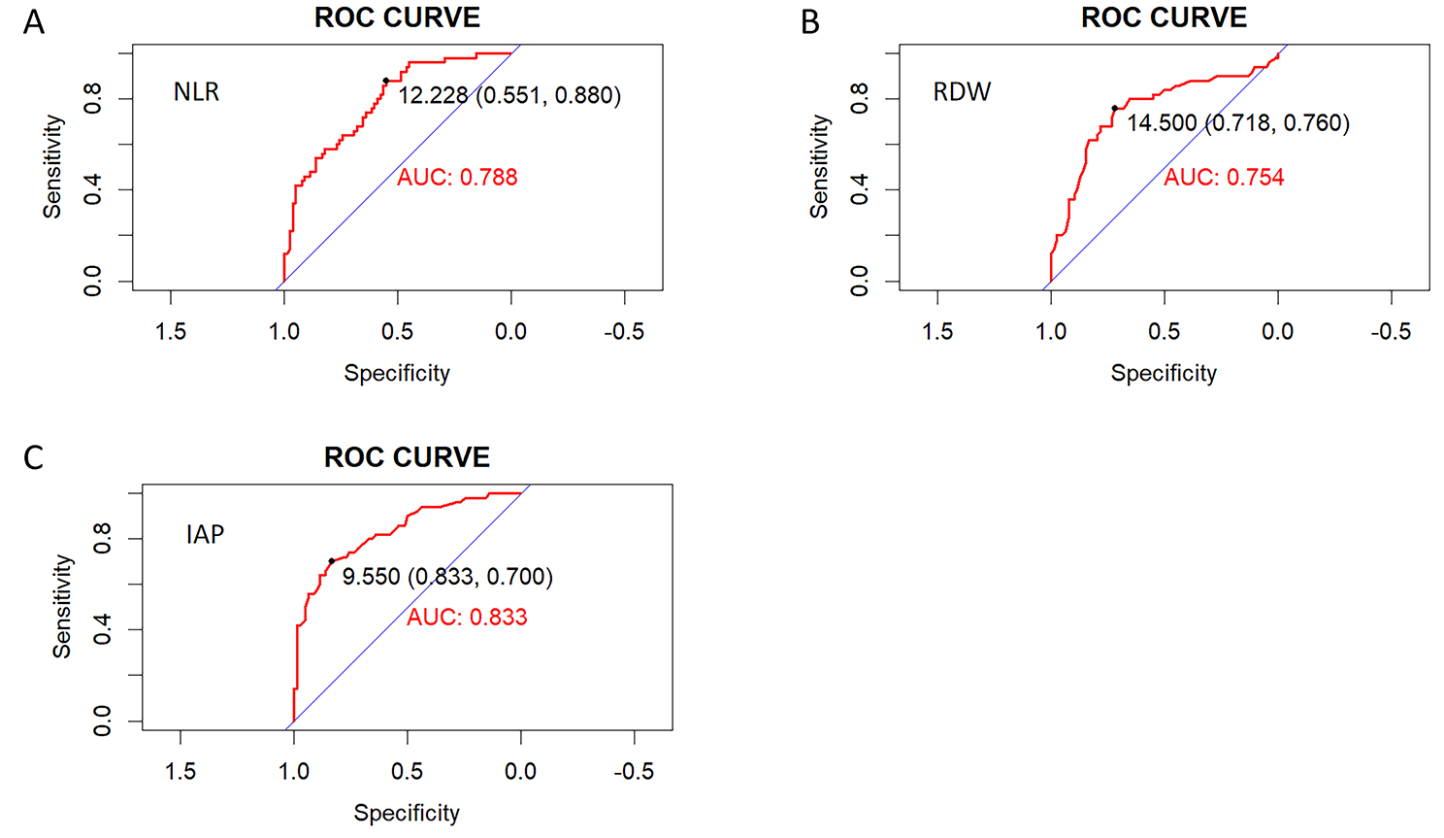
ROC curves of NLR, RDW and IAP predict SAPIP

The ROC curve of severity prediction constructed based on the potential factors of RDW, IAP and NLR were presented in Fig. 5. The respective areas under the curve (AUC) of RDW and NLR were 0.754 [95% confidence interval (CI) 0.662–0.846], 0.788 (95% CI 0.709–0.866). The cut-off value of RDW for predicting the occurrence of SAPIP was 14.5%, the sensitivity was 0.76, and the specificity was 0.718, the cut-off value of NLR for predicting the occurrence of SAPIP was 12.228, the sensitivity was 0.88, and the specificity was 0.551. Meanwhile, the area under the curve (AUC) of IAP was 0.833 [95% confidence interval (CI) 0.761–0.906], the cut-off value of IAP for predicting the occurrence of SAPIP was 9.55 mmHg, the sensitivity was 0.70, and the specificity was 0.833.

**Fig. 6:**
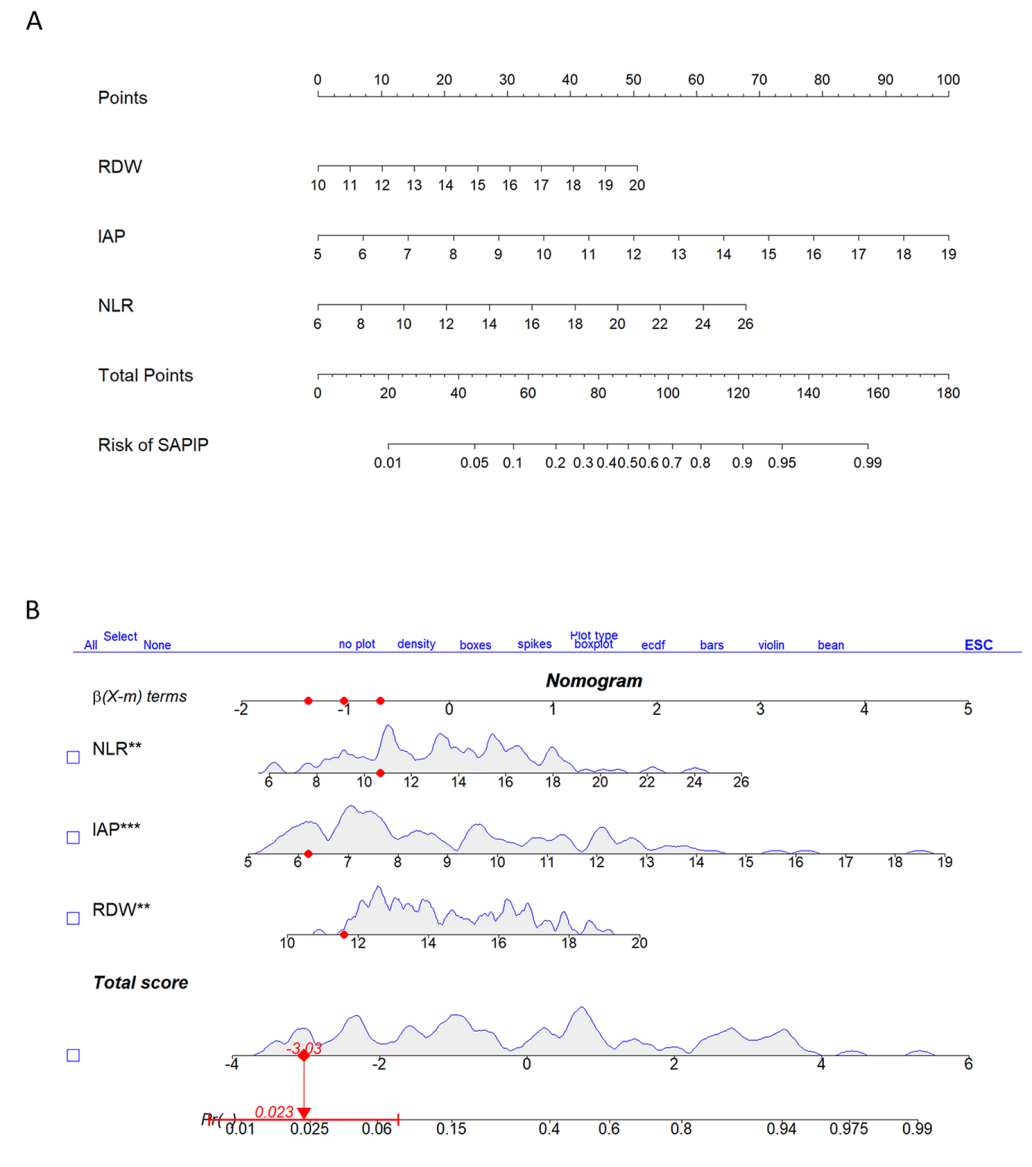
**A**: line-segment static nomograms. Scores for each level of every variable on the nomogram were determined by a vertical dot-line from that factor to the point scale. Therefore, a total point was obtained by summing all the values. Finally, the risk of SAPIP for each patient could be estimated based on the total points. **B**: Line-segment dynamic nomograms. In R studio, we can click on different characteristics to see the probability of SAPIP in patients with different characteristics

The predictive model was created according to the independent prediction-related factors identified by the multivariate logistic regression analysis and was showed as follows: SAPIP risk = -11.618 + 0.206 × NLR + 0.371 × RDW + 0.443 × IAP. Then, the severity prediction model was visualized by a nomogram. As shown in Fig. 6, we constructed two kinds of nomogram.

**Fig. 7:**
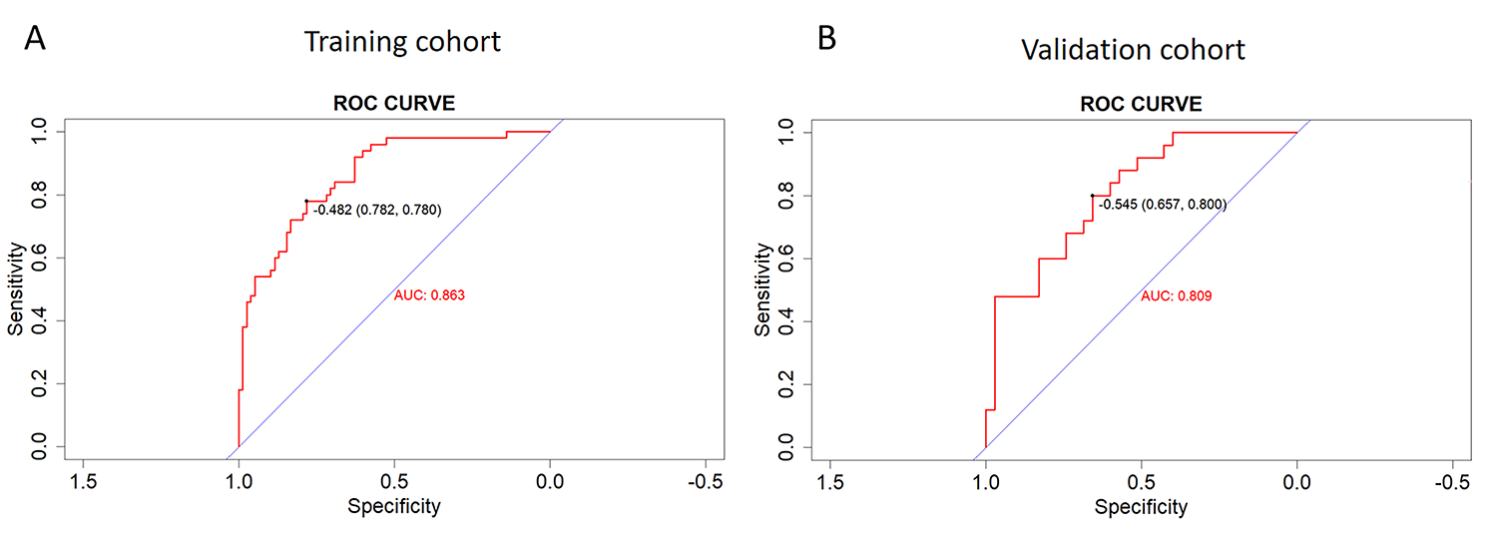
The ROC curves of the nomogram predicting SAPIP in the internal validation set (**A**) and external validation set (**B**)

**Fig. 8:**
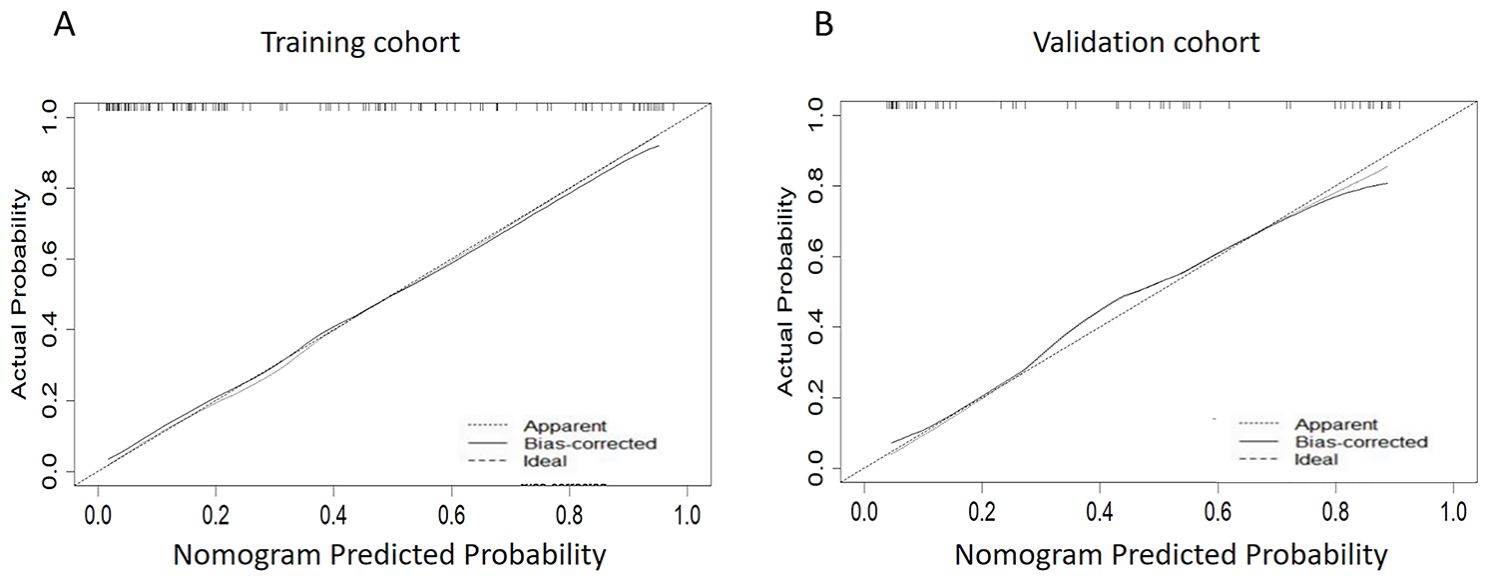
Calibration curve of poor prognosis prediction model in training cohort (**A**) and validation cohort (**B**)

In order to further check the validity of the prediction model, ROC curves were used to assess the discriminative property. The AUC of the predictive model in the training cohort (internal validation) was 0.863 (Fig. 7A ), and was 0.809 in the validation cohort (external validation) (Fig. 7B), indicating that the model has good discriminative ability. From the data in Fig. 8A and B, we knew that the calibration curves of internal (training cohort) and external (validation cohort) were very close to the 45°oblique line, showing that there was a great consistency between the predicted and actual results.

**Fig. 9:**
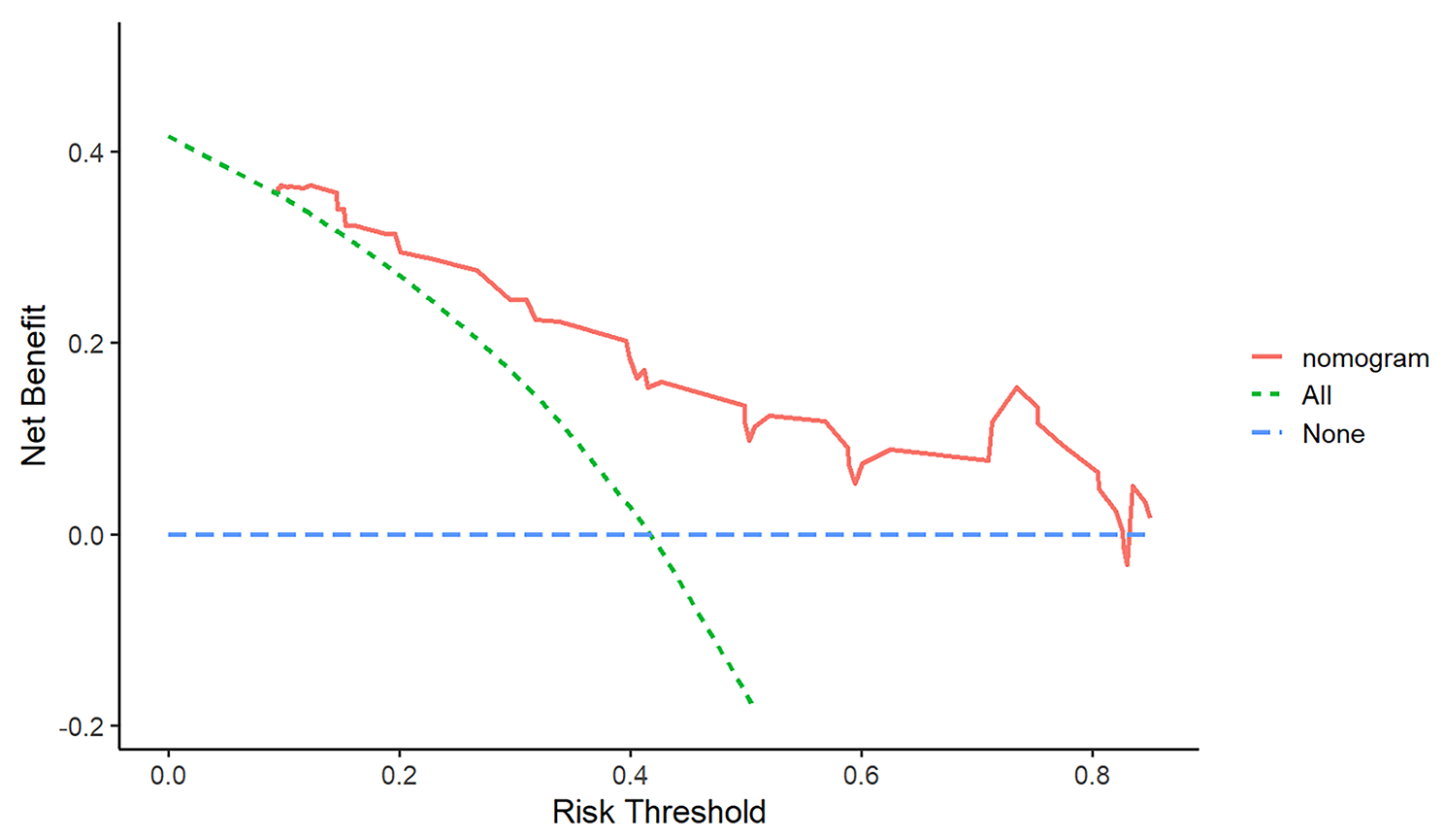
The decision curves of the nomogram predicting SAPIP

Finally, in order to evaluate the clinical usefulness and applicability of the model, we constructed a decision analysis curve. The decision curve shown in Fig. 9 indicated that the patients used this model can get more net benefit than the patients with complete intervention or no intervention at all. It means the model has potential clinical usefulness as a nomogram.

## Discussion

It is still difficult to early diagnose of SAP in the clinical treatment with AP patients [5, 23]. For APIP, early prediction of its severity is more difficult due to the complexity and specificity of pregnancy. At present, the measurement of hematuria amylase and enhanced CT scan are main techniques for AP diagnosis, but as every pancreatologist knows that the level of hematuria amylase is not in direct proportion to the severity of the disease [24, 25]. In addition, CT examination, especially enhanced CT scan, should be prudently selected for pregnant women, and uterine enlargement during pregnancy can lead to some changes in the anatomical position of abdominal organs, moreover, there are few specific predictive models for APIP, so it is relatively difficult to evaluate the severity of APIP. What’s more, APIP is a dangerous disease because of its rapid progression, once it escalates into SAP, it will be very harmful to both mother and fetus [26]. In a word, it is very useful to construct a new multi-factor clinical model to predict the severity of APIP, which will be helpful to deal with risk stratification and management of APIP.

RDW is a commonly used bio-marker to detect the severity of erythrocyte anisocytosis. The higher RDW indicates much more anisocytosis [27]. In addition, there are also many other studies showing that the abnormal elevation of RDW can predict the poor prognosis of patients with septic shock [28], acute myocardial infarction [29], and general trauma patients [30]. The predictive role of RDW in AP has received continuous attention [31, 32]. RDW has several advantages as a predictive marker. Firstly, RDW is a part of the blood routine test which is fairly inexpensive and is a routine test. Additionally, it can be accessed easily and its results can be obtained quickly. Some studies have proved that RDW is an independent risk factor related to the severity of AP [32, 33]. However, the predictive role of severity by RDW in APIP remains unclear. In our study, the higher level of RDW in SAPIP patients indicated that RDW maybe a potential predictor of SAPIP. After univariate and multivariate logistic regression analysis, we found RDW is an independent predictive factor of severity in APIP.

NLR is a widely-used marker of bodily inflammation, easily obtained from calculation of the parameters usually supplied in a full blood count report. NLR is the ratio of neutrophils to lymphocytes, which combines two different parts of the immune pathway and shows the balance between inflammatory activator neutrophils and inflammatory regulator lymphocytes. In addition, higher NLR values represent a more unbalanced inflammatory state [34]. There were some studies indicated that NLR is related to the severity of AP. Li et al. performed a retrospective study and found that NLR is the most significant biomarker of overall survival in the AP patient group [35]. Jeon et al. found that higher NLR value is closely associated with severe acute pancreatitis and organ failure [36]. In our study, we found the higher level of NLR in SAPIP patients indicated that NLR maybe a potential predictor of SAPIP. After univariate and multivariate logistic regression analysis, we determined that NLR is an independent predictive factor of severity in APIP.

Many studies have led to an increasing interest in the measurement of IAP as a indicator of prognostic or predictive severity in patients with acute pancreatitis [37–39]. Intra-abdominal hypertension (IAH) refers to a repeated pathological high IAP with the value of greater than or equal to 12 mmHg. It is commonly believed that IAH in AP with organ dysfunction indicates visceral oedema because of the inflammatory process [40]. More studies have been showed that it is important to monitor IAP in patients with AP as it can reflect severity and potential influence about management. Besides, studies also proved that IAP is related with organ dysfunction and mortality of pancreatitis [40, 41]. However, few studies have evaluate the value of IAP in APIP. Therefore, it remains unclear whether IAP can predict the severity of APIP. Our study determined that IAP is an independent severity predictor in APIP.

ROC curve analysis showed that RDW, IAP and NLR have a great predictive value in SAPIP. Moreover, in our study, a new predictive model consisting of three risk factors (RDW, IAP, NLR) was constructed. In addition, our new predictive model of severity in APIP based on the three risk factors (RDW, IAP and NLR) has good predictive value. Morever, we created a model-based prediction nomogram that provides a convenient metric for predicting the severity of APIP. The results can be obtained very quickly and accurately without replacing the numbers into the equation. Of note, the model showed great accuracy and consistency in both external and internal validation. The major advantage of our clinical model is that all variables can be obtained easily and quickly, providing a fast and reliable tool for clinical prediction of APIP. Yang’s team analyzed 190 cases of APIP and established the prediction model for moderately severe and severe acute pancreatitis based on lactate dehydrogenase, triglyceride, cholesterol, and albumin levels [42]; Sheng’s team constructed a nomogram for POF with APIP based on four indicators: lactate dehydrogenase, triglycerides, serum creatinine, and procalcitonin [43]. However, the above prediction models of APIP require more indicators, and some indicators cannot be obtained quickly, so patients’ conditions can’t be timely and accurately judged. Our blood indicators can be obtained from the blood routine, which takes only half an hour, and we can also quickly measure the IAP and get the value.About the clinical utility of the study findings, via this prediction model based on blood test and measurement of IAP, the doctors can early recognize APIP severity in the clinical work, which will be very helpful to deal with risk stratification and management of APIP.

Our study has several drawbacks. First off, our research was limited to a single-center retrospective design., so further multi-center studies are needed to support the results. Secondly, we only collected the data of 188 patients, further validation with a larger sample of data is required, and this is also our research plan to carry out in the future.

## Conclusion

In conclusion, we established and validated a new predictive nomogram model of severity according to RDW, IAP and NLR in APIP patients, which presents superior accuracy and accessibility. It is very useful to apply this model to stratify APIP patients for primary management and early intervention to improve prognosis.

## Data Availability

The datasets generated and/or analyzed during the current study are not publicly available because they contain the patients’ personal information, but are available from the corresponding author on reasonable request.

## Abbreviations

Alb: Albumin
ALT: Alanine aminotransferase
AP: Acute pancreatitis
APIP: Acute pancreatitis in pregnancy
BUN: Blood urea nitrogen
Ca: Calcium
DCA: Decision curve analysis
IAP: Intra-abdominal pressure
LDH: Lactate dehydrogenase
MSAP: Moderately severe acute pancreatitis
NLR: Neutrophil-lymphocyte ratio
RDW: Red cell distribution width
ROC: Curves:receiver operating characteristic curves
SAP: Severe acute pancreatitis
Scr: Serum creatinine
SD: Standard deviation
TC: Total cholesterol
TG: Triglyceride
